# Late-onset optic disc swelling-type Vogt–Koyanagi–Harada disease: diagnostic value of longitudinal multimodal imaging

**DOI:** 10.1186/s12348-026-00616-8

**Published:** 2026-06-23

**Authors:** Tetiana Goncharova, Sara Garcia-Hidalgo, Oscar Balaguer Sole, Armand Pairó-Salvador, Yann Bertolani, Eric Kirkegaard-Biosca, Laura Distefano

**Affiliations:** Ophthalmology Department, Vall d’Hebron University Hospital, Passeig de la Vall d’Hebron 129, Barcelona, 08035 Spain

**Keywords:** Vogt–Koyanagi–Harada disease, Bilateral optic disc edema, Glial fibrillary acidic protein astrocytopathy, Ovarian teratoma, Choroidal inflammation, Multimodal imaging

## Abstract

**Purpose:**

To describe an atypical late-onset presentation of Vogt–Koyanagi–Harada (VKH) disease initially interpreted as autoimmune glial fibrillary acidic protein astrocytopathy (GFAP-A) in association with an ovarian teratoma.

**Methods:**

A 59-year-old woman presented with bilateral optic disc edema, visual field defects, and headache following a flu-like illness. Comprehensive ophthalmologic, neurologic, and systemic evaluation was performed, including multimodal ocular imaging, cerebrospinal fluid (CSF) analysis with antibody testing, and neuroimaging. Clinical course and response to corticosteroid therapy were assessed longitudinally.

**Results:**

Initial findings, including CSF anti-GFAP positivity, lymphocytic pleocytosis, white matter lesions, and an ovarian teratoma, supported a diagnosis of GFAP-A. However, sequential multimodal imaging revealed diffuse, steroid-responsive choroidal inflammation with choroidal thickening, folds, mild subretinal fluid, hypocyanescent dark dots on indocyanine green angiography, fluorescein angiography pinpoints, and bilateral hot discs. Granulomatous anterior uveitis and subsequent choroidal depigmentation further supported VKH. Bilateral sensorineural hearing loss was also consistent with this diagnosis. The longitudinal ocular phenotype proved more consistent with VKH than with primary astrocytopathy-related optic neuropathy.

**Conclusions:**

Late-onset VKH may present predominantly with optic disc edema and central nervous system involvement, closely mimicking inflammatory neurologic disease. Recognition of subtle choroidal inflammation and integration of longitudinal multimodal imaging are essential to avoid misdiagnosis and guide appropriate immunosuppressive management.

## Introduction

Vogt–Koyanagi–Harada (VKH) disease is a multisystem autoimmune disorder targeting melanocyte-associated antigens in the uvea, meninges, inner ear, and skin. The classic presentation consists of bilateral granulomatous panuveitis with serous retinal detachments, frequently accompanied by headache, meningismus, and auditory symptoms. Cutaneous findings may appear later during the convalescent stage.

In older patients, however, VKH may manifest atypically, with bilateral optic disc edema predominating over exudative detachments. These incomplete forms may delay diagnosis and appropriate immunosuppressive therapy, increasing the risk of irreversible damage [1, 2].

Autoimmune glial fibrillary acidic protein astrocytopathy (GFAP-A) is a recently described inflammatory disorder characterized by GFAP-IgG antibodies in cerebrospinal fluid (CSF). It commonly presents as meningoencephalomyelitis, and optic disc swelling or optic neuritis may occur. More than 25% of cases are associated with neoplasms, particularly ovarian teratomas, suggesting a possible paraneoplastic mechanism.

We report a late-onset VKH case initially diagnosed as GFAP-A associated with an ovarian teratoma. However, the subsequent longitudinal ocular evolution ultimately proved more consistent with optic disc swelling (ODS)-type VKH disease [3, 4].

## Methods

We conducted a longitudinal clinical evaluation of a patient managed at a tertiary referral centre, integrating ophthalmologic, neurologic, and systemic findings.

Ophthalmic assessment included best-corrected visual acuity (BCVA), slit-lamp examination, funduscopy, optical coherence tomography (OCT), fluorescein angiography (FA), indocyanine green angiography (ICGA), and anterior segment OCT (AS-OCT).

Neurologic and systemic evaluation included CSF analysis with antibody testing, brain and spinal magnetic resonance imaging (MRI), brainstem auditory evoked potentials (BAEP), positron emission tomography–computed tomography (PET–CT), and targeted autoimmune and infectious investigations, including anti-AQP4, anti-MOG, and anti-GFAP antibodies.

Clinical course and response to corticosteroid therapy were assessed through serial clinical examinations and multimodal imaging.

### Case description

**Fig. 1:**
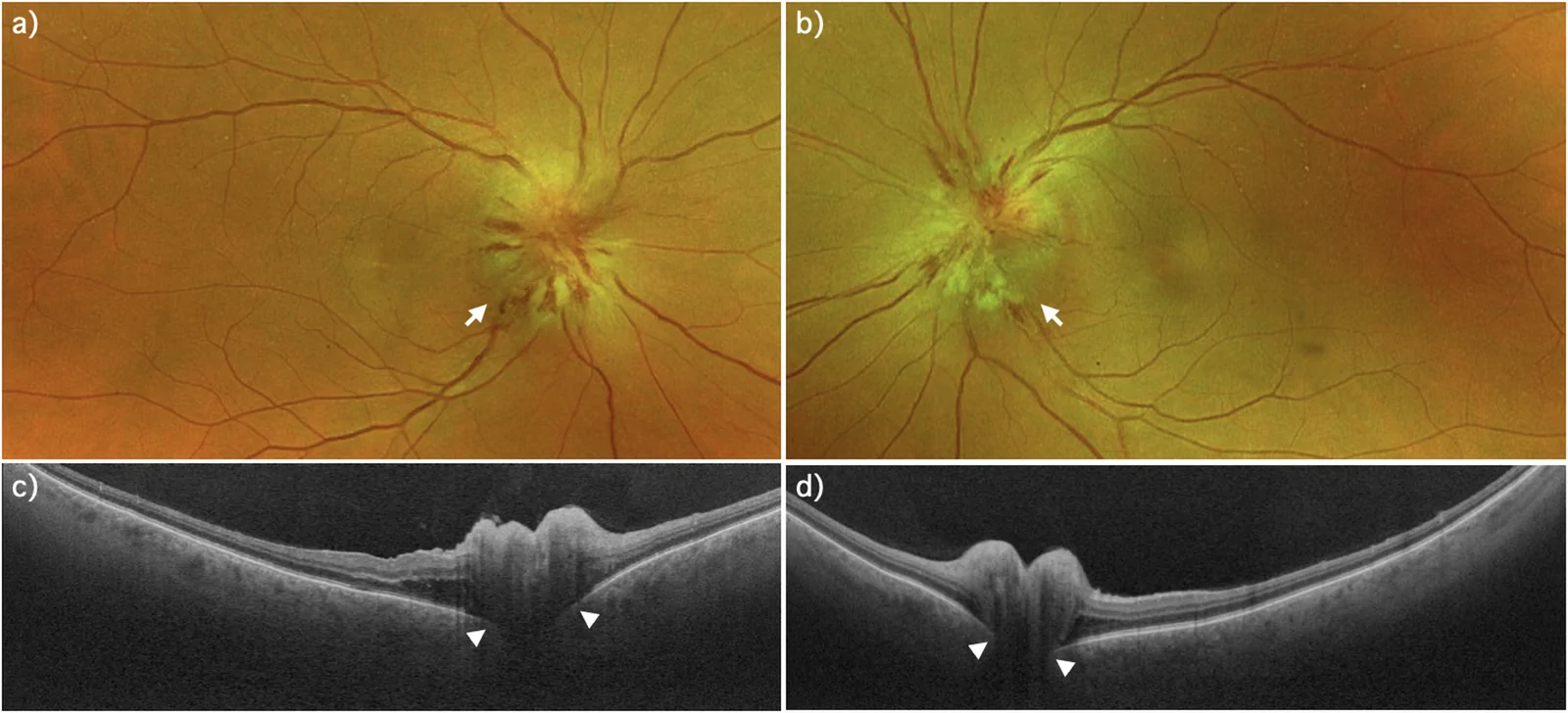
(**A** right eye, **B** left eye) Fundoscopy at the time of diagnosis reveals bilateral optic disc edema, accompanied by peripapillary hemorrhages and soft exudates (arrows). (**C** right eye, **D** left eye) Optical coherence tomography of the optic nerve head shows optic nerve swelling and mild peripapillary choroidal thickening, together with a concave-downward Bruch’s membrane opening angle (arrowheads)

A 59‑year‑old hypertensive woman presented with a superior altitudinal scotoma and photopsias in the right eye (RE), associated with holocranial frontal headache following a flu‑like illness two weeks earlier. BCVA was 20/50 (RE) and 20/30 (LE), with bilateral color vision impairment and a right relative afferent pupillary defect (RAPD). The anterior segment was unremarkable. Fundus examination revealed bilateral optic disc edema with peripapillary hemorrhages and soft exudates (Fig. 1A and B). OCT confirmed optic nerve swelling and mild peripapillary choroidal thickening (Fig. 1C and D). The patient was admitted under Neurology for further evaluation.

LP showed normal opening pressure and lymphocytic pleocytosis. Brain MRI demonstrated bilateral optic edema, a small lesion in the anterior intraorbital segment of the left optic nerve, multiple periventricular and subcortical white matter lesions, and T2-hyperintense lesions in the cerebellar white matter. Spinal MRI incidentally identified a lesion suggestive of a pelvic teratoma. Visual field (VF) testing showed superior and inferior arcuate defects in the RE and an inferior altitudinal defect in the LE.

During hospitalization, the patient reported subjective bilateral hearing loss, initially attributed to eustachian tube dysfunction after normal otorhinolaryngologic examination and pure-tone audiometry. A broad systemic, autoimmune, and infectious work‑up was negative, including anti‑AQP4, anti‑MOG, and antineuronal antibodies, and PET–CT revealed no additional lesions. When BCVA in the RE worsened to 20/200, intravenous methylprednisolone (1 g/day for 5 days) was administered, with improvement in headache, optic disc inflammation, and visual acuity. Given the combination of bilateral optic disc edema, meningeal inflammation, and a pelvic teratoma, CSF testing for anti-GFAP antibodies was performed and returned positive, leading to a diagnosis of autoimmune GFAP-A associated with ovarian teratoma.

**Fig. 2:**
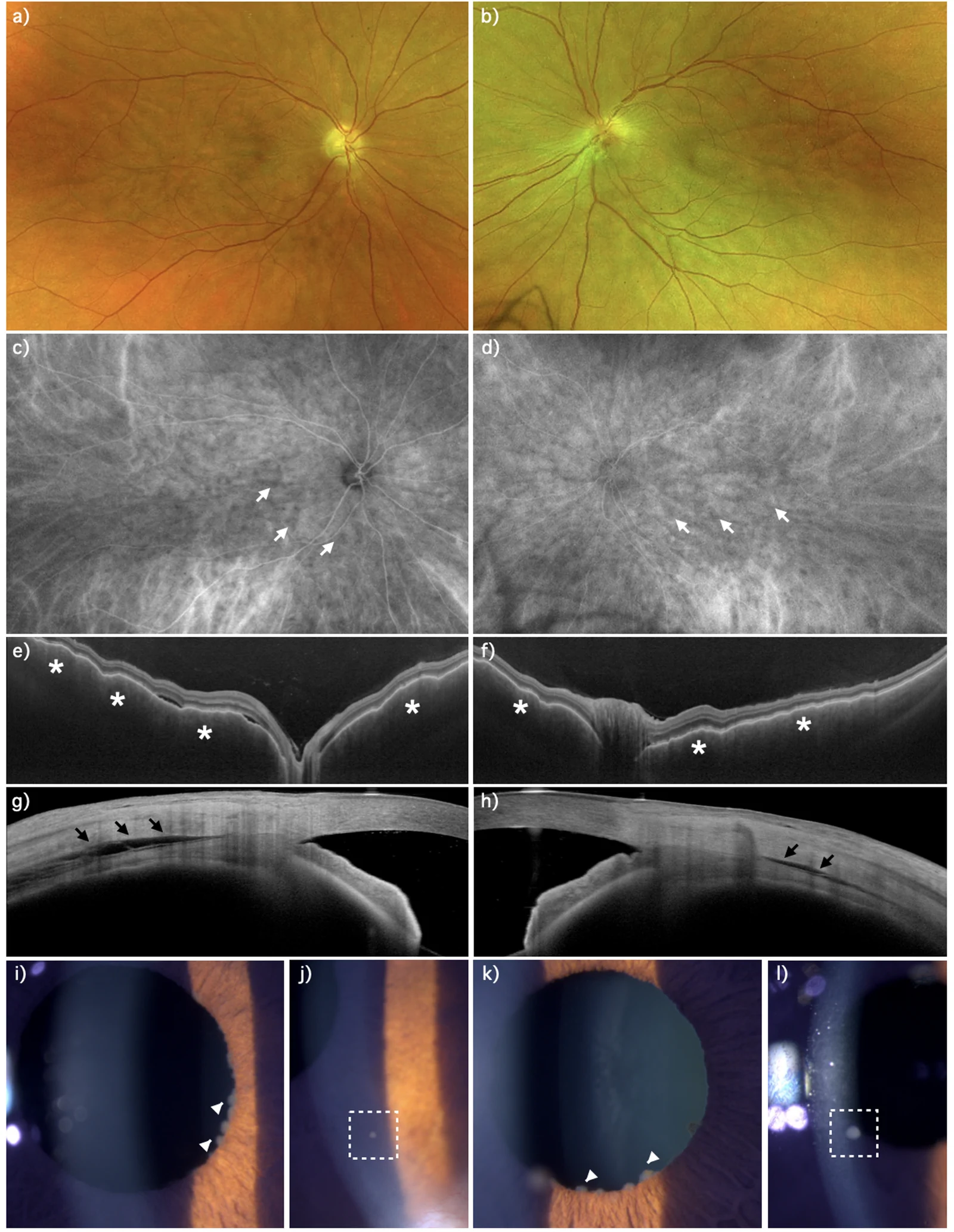
(**A** right eye, **B** left eye) Fundus examination of both eyes reveals multifocal diffuse choroidal infiltration with attenuation of choroidal vessels. (**C** right eye, **D** left eye) Indocyanine green angiography demonstrates diffuse hypocyanescent dark dots, predominantly in the posterior pole (white arrows) during late phases. (**E** right eye, **F** left eye) Macular OCT shows diffuse bilateral choroidal thickening (asterisks), accompanied by choroidal folds and subretinal fluid. Resolution of the papillary edema in the right eye is observed, although mild edema persists in the left eye. (**G** right eye, **H** left eye) Anterior segment OCT reveals minimal uveal effusion (black arrows). (**I** right eye, **K** left eye) Koeppe nodules, appearing as granular accumulations at the pupillary border (white arrowheads). (**J** right eye, **L** left eye) Isolated granulomatous precipitates are noted (white squares)

At one-month follow-up, optic disc edema had improved, revealing small, crowded discs (Fig. 2A and B). However, macular OCT demonstrated diffuse choroidal thickening in both eyes, with minimal subretinal fluid in the RE (Fig. 2E and F). FA showed delayed choroidal filling, bilateral hot discs, and diffuse late capillary leakage, while ICG identified numerous hypocyanescent dark dots (HDD) suggestive of choroidal infiltration (Fig. 2C and D). Ocular ultrasound excluded extraocular extension. Given this infiltrative choroidal pattern, choroidal lymphoma was considered in the differential diagnosis. Within weeks, granular deposits at the pupillary border (Koeppe nodules) (Fig. 2I and K), fine granulomatous keratic precipitates (KP) (Fig. 2J and L), anterior chamber cells, and elevated intraocular pressure developed bilaterally, and AS-OCT showed mild uveal effusion (Fig. 2G and H). An anterior chamber paracentesis was performed. Flow cytometric analysis of the aqueous humor revealed a predominance of CD4 + T lymphocytes, with no detectable B-cell or aberrant populations [5]. The IL-10/IL-6 ratio was 0.02, supporting an inflammatory process. At that stage, the ocular findings were initially interpreted as a sarcoid-like inflammatory response associated with GFAP-A.

**Fig. 3:**
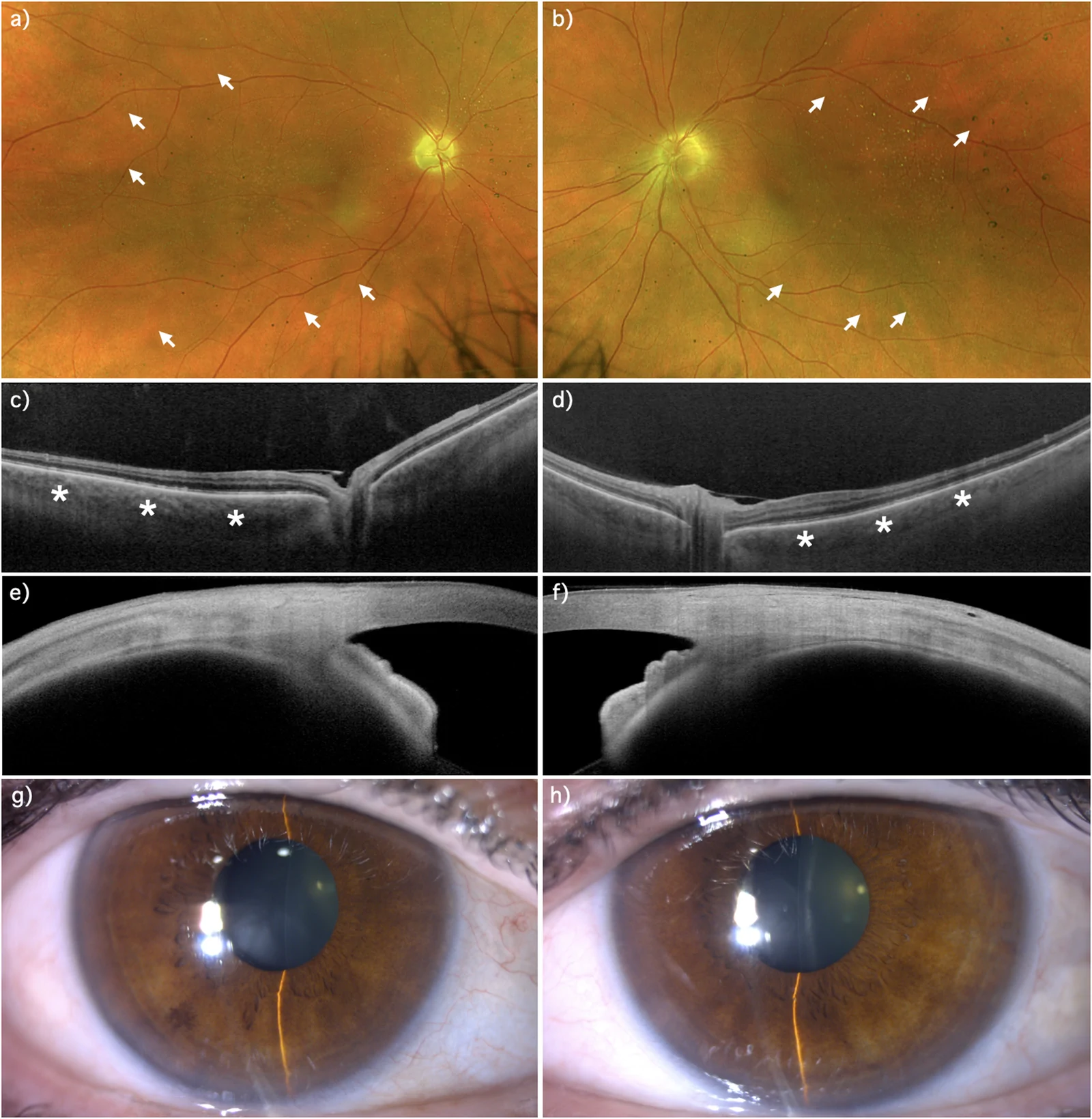
(**A** right eye, **B** left eye) Fundus examination of both eyes showing improved choroidal infiltration, with visualization of choroidal vessels (white arrows). (**C** right eye, **D** left eye) Macular OCT reveals a reduction in choroidal thickening (white asterisks), along with the resolution of choroidal folds, subretinal fluid, and papillary edema. (**E** right eye, **F** left eye) Anterior segment OCT reveals normalization of the anterior segment, with resolution of uveal effusion. (**G** right eye, **H** left eye) Anterior segment photographs show the disappearance of Koeppe nodules and granulomatous precipitates

Topical dexamethasone every two hours with weekly taper led to excellent anterior segment response, and oral prednisone 60 mg/day with biweekly tapering was initiated for posterior involvement, resulting within one month in marked reduction of choroidal thickness and complete resolution of choroidal folds (Fig. 3). During tapering, anterior‑predominant relapses occurred, with recurrent Koeppe nodules and granulomatous KP that responded to intensified topical corticosteroids.

**Fig. 4:**
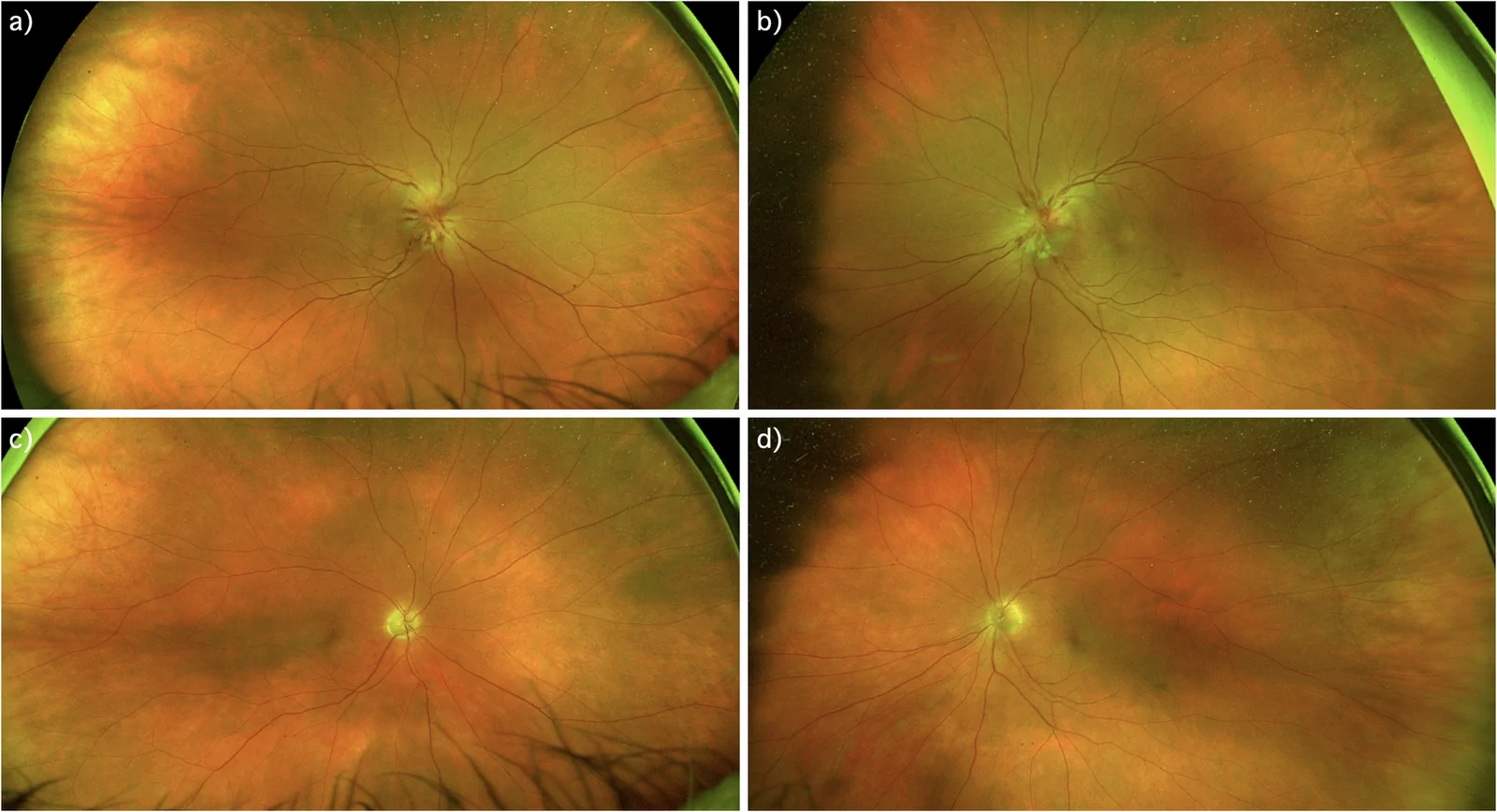
(**A** right eye, **B** left eye) Fundus photographs at the time of diagnosis showing bilateral optic disc edema. (**C** right eye, **D** left eye) Follow-up fundus images six months later reveal mild choroidal depigmentation, predominantly affecting the inferior and nasal retina

At six months, brain MRI showed persistent small white matter lesions, resolution of optic disc edema, and a new focal T2-hyperintense lesion in the left cerebellar peduncle. Mild choroidal depigmentation on widefield fundus photography (Fig. 4) prompted a comprehensive reassessment, which identified a preceding viral prodrome, bilateral visual impairment, and bilateral sensorineural hearing loss confirmed by BAEP. Additional findings included diffuse, steroid-responsive choroidal inflammation, initially manifesting as posterior segment involvement — choroidal thickening, folds, and mild subretinal fluid — followed by anterior segment granulomatous signs, including Koeppe nodules and KP.

**Fig. 5:**
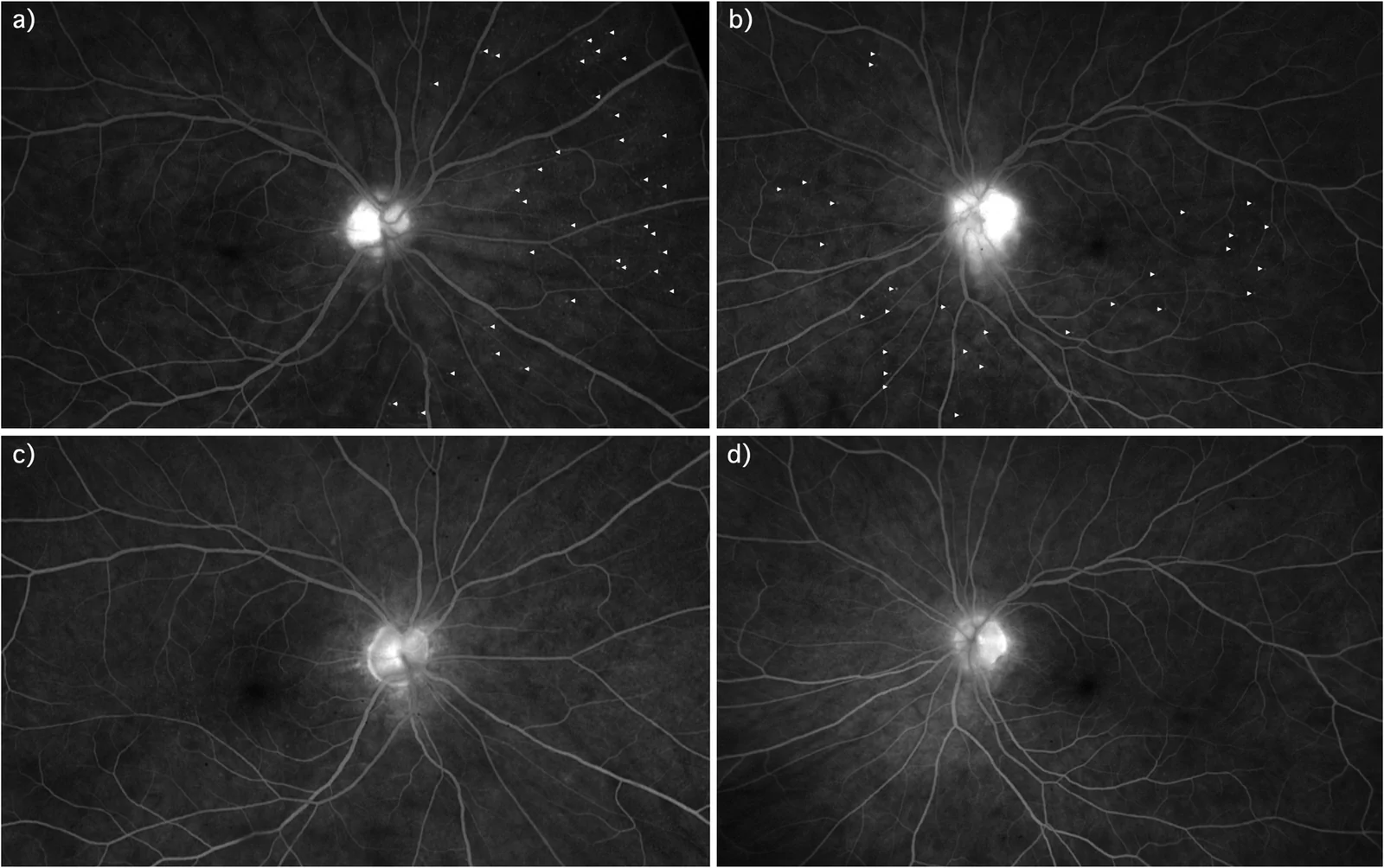
(**A** right eye, **B** left eye) Fluorescein angiography in the late phase revealed subtle hyperfluorescent dots in the peripapillary region suggestive of VKH-like pinpoints (white arrowheads), diffuse capillaritis, and bilateral optic disc hyperfluorescence (“hot discs”). (**C** right eye, **D** left eye) Follow-up fluorescein angiography four months after initiating oral corticosteroid therapy shows resolution of the pinpoints and optic disc hyperfluorescence

Retrospective review of widefield OCT at presentation and at two months demonstrated peripapillary choroidal thickening with a fuzzy choroidal appearance, disruption of the ellipsoid and interdigitation zones, internal limiting membrane fluctuations, blurred choroidal contour, and small hyperreflective deposits above the retinal pigment epithelium— features previously described in VKH. FA revealed subtle peripapillary hyperfluorescent dots resembling VKH pinpoints, diffuse capillaritis, and bilateral hot discs, all of which resolved after corticosteroid therapy (Fig. 5).

One year later, and after multidisciplinary surgical planning due to the complexity of the procedure, the patient underwent right adnexectomy combined with infraumbilical preperitoneal hernioplasty. Histopathological examination revealed a mature cystic teratoma with extensive ischemic necrosis, without identifiable neural tissue in the viable non-necrotic areas. The hernia sac showed fibroadipose tissue with focal chronic inflammation.

Six months after surgery, while receiving oral prednisone 10 mg daily and topical dexamethasone every 48 h, the patient continued to experience recurrent episodes of anterior segment inflammation, together with persistent HDD on ICGA. BCVA was 1.0 in both eyes. Control VF testing demonstrated incomplete arcuate defects in the RE and an inferonasal defect in the LE. Additionally, optic disc and macular OCT revealed thinning of the retinal nerve fiber layer and ganglion cell layer in the RE, with relative preservation of both layers in the LE.

Overall, the findings were deemed most consistent with ODS-type VKH disease in an older patient, initially presenting with bilateral optic disc edema and presumed non‑arteritic anterior ischemic optic neuropathy (NA-AION), likely related to disc crowding and vascular comorbidities during the early phase of juxtapapillary choroidal thickening. The patient is currently being evaluated for initiation of biologic therapy in order to achieve sustained inflammatory control and as a corticosteroid-sparing strategy.

## Discussion

This case illustrates a late-onset presentation of incomplete VKH disease according to the Revised Diagnostic Criteria, in which bilateral optic disc swelling with minimal or absent serous retinal detachment, concomitant central nervous system (CNS) lesions, and a paraneoplastic context closely mimicked autoimmune GFAP-A. Beyond its diagnostic complexity, this case expands the recognized spectrum of ODS-type VKH and highlights the importance of longitudinal multimodal imaging when neuro-ophthalmic and inflammatory phenotypes overlap.

ODS-type VKH is increasingly recognized, particularly in older patients, and is characterized by optic disc edema disproportionate to the degree of exudative retinal detachment, often leading to an initial diagnosis of ischemic optic neuropathy, papillitis, or optic neuritis. In the series by Nakao et al. [2], ODS-type VKH was identified in 27.6% of eyes and was associated with older age and crowded optic disc morphology. Our patient closely fit this profile. The combination of small crowded discs and vascular comorbidities likely amplified the effects of early juxtapapillary choroidal inflammation, predisposing to impaired short posterior ciliary circulation and axoplasmic flow stasis, thereby explaining the RAPD, dyschromatopsia, and altitudinal VF defects resembling NA-AION [6, 7].

**Fig. 6:**
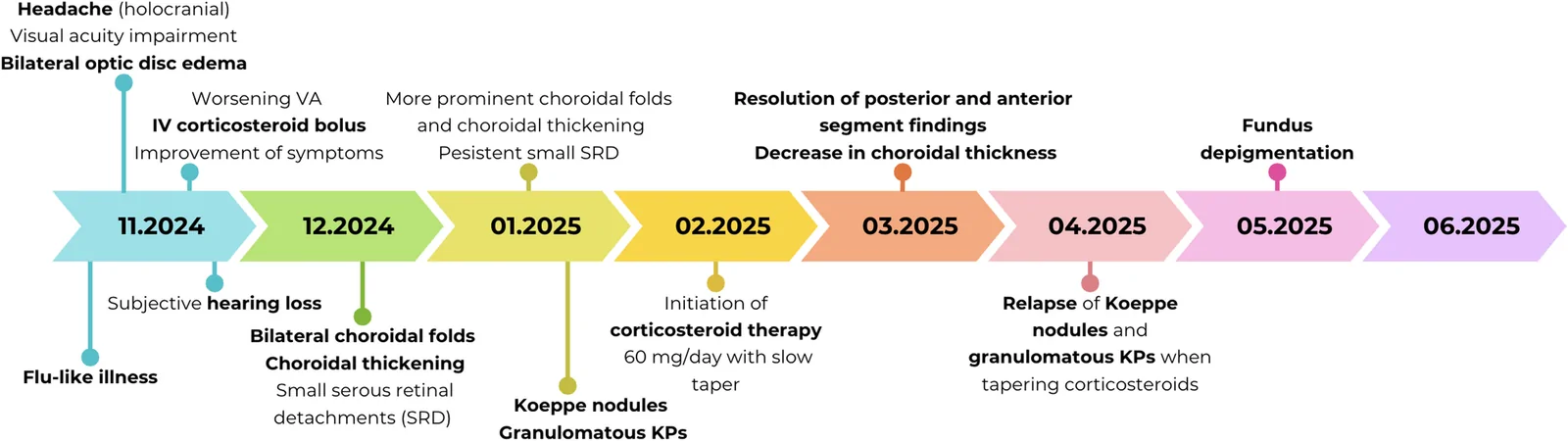
Chronological timeline summarizing the patient’s clinical evolution leading to the diagnosis of VKH disease. Following a flu-like illness, the patient developed bilateral optic disc edema, visual impairment, hearing symptoms, and progressive multimodal imaging findings of diffuse choroiditis with granulomatous anterior uveitis. The subsequent evolution toward fundus depigmentation and recurrent inflammation during corticosteroid tapering ultimately supported a VKH inflammatory phenotype

Importantly, clinically significant choroidal inflammation is present in ODS-type VKH despite subtle or absent retinal detachment, making choroid-focused multimodal imaging crucial for diagnosis [8, 9]. In our case, OCT already demonstrated juxtapapillary and peripapillary choroidal thickening at presentation, an early sign more consistent with VKH choroiditis than with the optic nerve-predominant ocular involvement typically described in GFAP-A. Serial multimodal imaging proved decisive: OCT examinations demonstrated steroid-responsive diffuse choroidal thickening and folds with mild subretinal fluid; ICGA revealed numerous HDD; and FA demonstrated subtle VKH-like pinpoints and bilateral hot discs [9]. Collectively, these findings were highly supportive of diffuse stromal choroidal inflammation, the central pathophysiological hallmark of VKH disease. During follow-up, the patient subsequently developed granulomatous anterior uveitis with KP and Koeppe nodules, followed by progressive choroidal depigmentation, thereby completing a characteristic VKH inflammatory trajectory that is difficult to reconcile with primary GFAP astrocytopathy (Fig. 6).

The coexistence of an ovarian teratoma, CSF GFAP-IgG positivity, lymphocytic pleocytosis, and multifocal white matter lesions initially raised the possibility of autoimmune GFAP-A, a steroid-responsive meningoencephalomyelitis in which optic papillitis and optic neuritis may occur and where ovarian teratomas have been reported in a subset of cases [3]. However, several clinicoradiological features progressively weakened this interpretation. Most importantly, the patient lacked the characteristic MRI pattern of GFAP-A, particularly the linear radial perivascular enhancement and longitudinally extensive myelitis considered among the strongest discriminators of true GFAP-A [3, 10] (Table 1). Furthermore, VKH itself is a systemic uveomeningoencephalitic disorder and may exhibit nonspecific CNS MRI abnormalities, including scattered periventricular or subcortical white matter lesions, brainstem involvement, and meningeal enhancement, thereby mimicking multiple sclerosis and other neuroinflammatory conditions [11]. Accordingly, the small periventricular, subcortical, and cerebellar lesions observed in our patient may reasonably be interpreted as compatible with VKH-related CNS involvement, particularly in the context of concurrent CSF pleocytosis and highly VKH-defining ocular imaging findings, rather than necessarily indicating a separate primary CNS inflammatory disease.

**Table 1:** Key differentiating features between VKH and autoimmune GFAP-A

Feature	VKH Disease	Anti-GFAP Astrocytopathy
Primary Target	Melanocytes in uveal tract, meninges, inner ear, skin	Astrocytes (GFAP-expressing cells)
Clinical Phenotypes	Prodromal → acute panuveitic → convalescent → chronic/recurrent phases	Subacute meningoencephalomyelitis, meningoencephalitis, optic neuritis/papillitis
Hallmark diagnostic test	Clinical diagnostic criteria supported by multimodal ocular imaging	CSF GFAP-IgG positivity (preferred over serum) with compatible clinicoradiologic syndrome
Neurological features	Headache, meningismus, lymphocytic meningitis, tinnitus, hearing loss	Headache, fever, encephalopathy, limb weakness/numbness, consciousness disturbance, tremor, seizures, urinary retention
CSF Findings	Lymphocytic pleocytosis (aseptic meningitis) can be present	Lymphocytic pleocytosis, elevated protein, CSF eosinophilia possible
Dermatologic/Auditory features	Poliosis, vitiligo, alopecia, hearing impairment common	Not typical; may occur but far less characteristic than VKH
Brainstem auditory evoked potentials	Peripheral (cochlear/endocochlear) sensorineural hearing loss	Not characteristic, not a defining feature
Ocular involvement		
*Ocular symptoms*	Bilateral blurred vision, floaters, photophobia and ocular pain	Asymptomatic or presenting with mild vision loss
*Fundus examination findings*	**Acute phase**: optic disc hyperemia and edema, bilateral serous retinal detachments, panuveitis **Chronic phase**: sunset glow fundus, subretinal fibrosis	Bilateral optic disc edema, mild vitritis possible
*Macular Optical Coherence Tomography (OCT)*	Subretinal fluid with septated appearance, choroidal thickening, RPE undulations, outer retinal disruption	Typically normal, no primary macular/choroidal disease. Possible mild inner retinal edema if optic disc edema is severe, but no choroidal thickening
*Fluorescein Angiography (FA)*	**Acute**: multiple early hyperfluorescent points, diffuse leakage from choroidal inflammation, progressive pooling of dye in subretinal space, disc hyperfluorescence (hot disc) **Chronic**: window defects (RPE atrophy), late staining of fibrotic scars	Optic disc hyperfluorescence/leakage, no primary retinal or RPE leakage, no macular pooling; normal choroidal phase
*Indocyanine Green Angiography (ICG)*	Hypofluorescent dark dots in early/mid-phases, diffuse choroidal inflammation	Not typically performed; not a diagnostic feature No primary choroidal involvement; normal ICGA expected
MRI findings		
*Characteristic pattern*	Nonspecific white matter lesions when brain MRI is performed; may mimic MS	Linear perivascular radial enhancement perpendicular to ventricles (45%)
*Leptomeningeal enhancement*	Can occur	Common (30%)
*Periependymal enhancement*	Not typical	Present
*Brainstem involvement*	Rare/nonspecific: reported brainstem/peduncle lesions	Common (T2/FLAIR hyperintensities) Bilateral middle cerebellar peduncle hyperintensities
*Spinal cord*	Very rare	Longitudinally extensive myelitis (49%), central gray matter involvement
*Basal ganglia/thalamus*	Not characteristic	T2 hyperintensities common (especially in children)

Side-by-side comparison of the main target tissue, typical clinical phenotypes, CSF profile, hallmark neuroimaging patterns, and multimodal ocular imaging findings that aid in distinguishing VKH from autoimmune GFAP-A

Increasing evidence indicates that GFAP-IgG is not invariably disease-specific and may occur in other inflammatory or paraneoplastic contexts [12, 13]. In the present case, the available clinicopathological evidence supports a broader teratoma-associated immune dysregulation rather than a classic antigen-specific paraneoplastic astrocytopathy. Although ovarian teratomas are well-recognized triggers of highly specific paraneoplastic syndromes, several findings argue against a primary teratoma-driven GFAP-A: the absence of the characteristic MRI pattern of GFAP-A, the subsequent evolution toward a highly VKH-defining ocular phenotype, and the pathological findings after oophorectomy, which revealed a mature cystic teratoma with extensive ischemic necrosis but no identifiable neural tissue within viable areas. Because neural differentiation within teratomas has been proposed as a key trigger for paraneoplastic neuroimmunological syndromes, the absence of recognizable neural tissue further weakens the hypothesis of a classic teratoma-driven GFAP-A. Instead, the teratoma may have acted as a nonspecific immunological catalyst, facilitating loss of immune tolerance and amplifying a systemic dysimmune response that ultimately manifested predominantly as VKH. Notably, the disease onset shortly after a preceding flu-like illness raises the additional possibility that an external inflammatory trigger contributed to the activation or unmasking of this immune dysregulation in a predisposed immunological context. Furthermore, persistent ocular inflammatory activity six months after tumor resection further argues against a purely teratoma-dependent inflammatory process. Within this framework, CSF GFAP-IgG positivity may represent a secondary or epiphenomenal marker of immune activation rather than the principal driver of the clinical syndrome.

From a practical standpoint, this case underscores several important diagnostic lessons. First, VKH should remain within the differential diagnosis of bilateral optic disc edema in older adults, particularly when multimodal imaging reveals even subtle evidence of choroidal inflammation or when audiovestibular symptoms are present. Second, the case highlights the critical value of longitudinal ophthalmic follow-up and serial multimodal imaging in atypical neuro-ophthalmic presentations. Finally, our findings emphasize the limitations of interpreting isolated serological or neuroradiological biomarkers outside the broader clinical context. In inflammatory disorders with overlapping neuro-ophthalmic manifestations, the evolving ocular phenotype may ultimately prove more diagnostically informative than isolated serological or neuroradiological findings.

## Data Availability

No datasets were generated or analysed during the current study.

## Abbreviations

AS-OCT: Anterior segment optical coherence tomography
BAEP: Brainstem auditory evoked potentials
BCVA: Best-corrected visual acuity
CNS: Central nervous system
CSF: Cerebrospinal fluid
FA: Fluorescein angiography
GFAP: Glial fibrillary acidic protein
GFAP-A: Glial fibrillary acidic protein astrocytopathy
ICGA: Indocyanine green angiography
IL: Interleukin
KP: Keratic precipitates
LE: Left eye
MRI: Magnetic resonance imaging
NA-AION: Non-arteritic anterior ischaemic optic neuropathy
OCT: Optical coherence tomography
PET–CT: Positron emission tomography–computed tomography
RAPD: Relative afferent pupillary defect
RE: Right eye
VF: Visual field
VKH: Vogt–Koyanagi–Harada disease
